# Supplementary material for: Performance of prediction models for short-term outcome in COVID-19 patients in the emergency department: a retrospective study
Source: Ann Med. 2021 Feb 25;53(1):402–9. doi: 10.1080/07853890.2021.1891453 (PMC7919920; doi:10.1080/07853890.2021.1891453)
Supplement: Supplemental Material [file IANN_A_1891453_SM7915.docx]

**Supplementary Table 1**. Comparison of the AUCs of included prediction models in the subgroup analysis (patients without treatment restrictions only, n = 261).

| **Prediction model** | **30-day mortality**  **(AUC, 95% CI)** | **Difference with RISE UP**  **(p value) ^a^** | **14-day mortality**  **(AUC, 95% CI)** | **Difference with RISE UP**  **(p value) ^a^** | **Composite endpoint (mortality and/or MCU/ICU admission)**  **(AUC, 95% CI)** | **Difference with RISE UP (p value) ^a^** |
| --- | --- | --- | --- | --- | --- | --- |
| RISE UP | 0.84 (0.76-0.91) | reference | 0.82 (0.72-0.92) | reference | 0.81 (0.75-0.87) | reference |
| 4C Mortality score | 0.85 (0.77-0.94) | 0.617 | 0.84 (0.75-0.93) | 0.644 | 0.77 (0.71-0.84) | 0.148 |
| CURB-65 | 0.75 (0.64-0.86) | 0.039 | 0.75 (0.62-0.87) | 0.156 | 0.65 (0.58-0.73) | <0.001 |
| MEWS | 0.63 (0.49-0.76) | <0.001 | 0.58 (0.40-0.75) | <0.001 | 0.78 (0.71-0.85) | 0.785 |
| REMS | 0.76 (0.67-0.86) | 0.154 | 0.80 (0.73-0.87) | 0.765 | 0.72 (0.65-0.79) | 0.020 |
| abbMEDS | 0.78 (0.69-0.86) | 0.095 | 0.78 (0.68-0.89) | 0.427 | 0.72 (0.65-0.78) | 0.007 |
| SOFA | 0.73 (0.61-0.84) | 0.034 | 0.74 (0.59-0.89) | 0.245 | 0.79 (0.73-0.86) | 0.877 |
| APACHE II | 0.69 (0.56-0.82) | 0.036 | 0.77 (0.64-0.90) | 0.578 | 0.68 (0.61-0.76) | 0.005 |
| CALL score | 0.75 (0.66-0.84) | 0.073 | 0.77 (0.66-0.87) | 0.359 | 0.67 (0.59-0.74) | <0.001 |
| ACP index | 0.73 (0.64-0.82) | 0.008 | 0.73 (0.63-0.83) | 0.053 | 0.68 (0.61-0.75) | <0.001 |
| Host risk factor score | 0.70 (0.60-0.79) | 0.022 | 0.69 (0.57-0.82) | 0.100 | 0.63 (0.59-0.71) | <0.001 |

4C, Coronavirus Clinical Characterisation Consortium; abbMEDS, abbreviated Mortality Emergency Department Sepsis; ACP, Age C-reactive protein; APACHE II, Acute Physiology and Chronic Health Evaluation II; AUC, area under the curve; CALL, Comorbidity Age Lymphocyte LDH; CI, confidence interval; CURB-65, Confusion Urea Respiration Blood pressure; ICU, intensive care unit; MCU, medium care unit; MEWS, Modified Early Warning Score; REMS, Rapid Emergency Medicine Score; RISE UP, Risk Stratification in the Emergency Department in Acutely Ill Older Patients; SOFA, Sepsis-related Organ Failure Assessment.

^a^ Comparison of the AUC of the prediction model with the AUC of the RISE UP score.

**Supplementary table 2**. Vital sign and laboratory results of study sample (n = 403).

|  | **Reference values** | **Study sample** | **Missing values,**  **n(%)** |
| --- | --- | --- | --- |
| Vital signs |  |  |  |
| HR, median (IQR), bpm |  | 90 (78-105) | 4 (1.0) |
| MAP, median (IQR), mmHg |  | 100 (90-108) | 3 (0.7) |
| RR, median (IQR), per minute |  | 20 (16-25) | 0 (0.0) |
| O2 saturation, median (IQR), % |  | 95 (92-97) | 0 (0.0) |
| Temperature, median (IQR), °C |  | 37.7 (37.0-38.5) | 2 (0.5) |
| Abnormal GCS (< 15), n% |  | 48 (11.9) | 0 (0.0) |
|  |  |  |  |
| Laboratory results |  |  |  |
| Hemoglobin, mean (SD), mmol/L | ♀ 7.5 – 10.0  ♂ 8.5 – 11.0 | 8.3 (1.3) | 7 (1.7) |
| Hematocrite, mean (SD), % | ♀ 0.36 – 0.48  ♂0.41 – 0.52 | 0.39 (0.05) | 7 (1.7) |
| Leukocytes, median (IQR), x 10^9^/L | 4.0 – 10.0 | 7.0 (5.3-9.5) | 6 (1.5) |
| Lymphocytes, median (IQR), x 10^9^/L | 1.1 – 4.0 | 0.8 (0.6-1.2) | 71 (17.6) |
| Thrombocytes, mean (SD), x 10^9^/L | 150 – 400 | 224 (100) | 17 (4.2) |
| pH, mean (SD) | 7.35 – 7.45 | 7.46 (0.08) | 144 (35.7) |
| pO_2,_ mean (SD), kPa | 8.7 – 13.1 | 10.4 (3.7) | 144 (35.7) |
| Sodium, mean (SD), mmol/L | 135 – 145 | 136 (4) | 12 (3.0) |
| Potassium, mean (SD), mmol/L | 3.5 – 5.0 | 4.23 (0.69) | 17 (4.2) |
| BUN, median (IQR), mmol/L | 3.5 – 7.5 | 6.2 (4.5-9.7) | 19 (4.7) |
| Creatinine, median (IQR), µmol/L | 70 – 110 | 87 (71-114) | 12 (3.0) |
| Bilirubin, median (IQR), µmol/L | < 21 | 8.7 (6.3-12.3) | 39 (9.7) |
| LDH, median (IQR), U/L | ♀< 247 ♂< 248 | 328 (239-451) | 21 (5.2) |
| Albumin, median (IQR), g/L | 35 – 50 | 31.9 (28.3-35.2) | 53 (13.2) |
| CRP, median (QRS), mg/L | < 10 | 69 (29-130) | 16 (4.0) |

BUN, blood urea nitrogen; CRP, C-reactive protein;GCS, Glasgow coma scale; HR, heart rate;IQR, interquartile range; LDH, lactate dehydrogenase; MAP, mean arterial pressure;RR, respiratory rate; SD, standard deviation.
